# A Prospective Evaluation of the Acute Effects of High Altitude on Cognitive and Physiological Functions in Lowlanders

**DOI:** 10.3389/fphys.2021.670278

**Published:** 2021-04-28

**Authors:** Marika Falla, Costanza Papagno, Tomas Dal Cappello, Anna Vögele, Katharina Hüfner, Jenny Kim, Elisabeth M. Weiss, Bernhard Weber, Martin Palma, Simona Mrakic-Sposta, Hermann Brugger, Giacomo Strapazzon

**Affiliations:** ^1^Institute of Mountain Emergency Medicine, Eurac Research, Bolzano, Italy; ^2^Center for Mind/Brain Sciences CIMeC, University of Trento, Rovereto, Italy; ^3^Department of Psychology, University of Milano-Bicocca, Milan, Italy; ^4^Department of Psychiatry, Psychotherapy and Psychosomatics, Division of Psychiatry II, Medical University of Innsbruck, Innsbruck, Austria; ^5^Institute for Exercise and Environmental Medicine, Texas Health Presbyterian Hospital, Dallas, TX, United States; ^6^Department of Psychology, University of Innsbruck, Innsbruck, Austria; ^7^Department of Psychology, University of Graz, Graz, Austria; ^8^Institute of Clinical Physiology, National Research Council (CNR), Milan, Italy

**Keywords:** altitude, cognitive functions, speed-processing, decision-making, attention

## Abstract

Cognitive function impairment due to high altitude exposure has been reported with some contradictory results regarding the possible selective cognitive domain involvement. We prospectively evaluated in 36 lowlanders, exposed for 3 consecutive days to an altitude of 3,269 m, specific cognitive abilities (attention, processing speed, and decision-making) required to safely explore the mountains, as well as to work at altitude. We simultaneously monitored the physiological parameters. Our study provides evidence of a reduced processing speed in lowlanders when exposed to altitude in the first 24 h. There was a fairly quick recovery since this impairment was no more detectable after 36 h of exposure. There were no clinically relevant effects on decision-making, while psychomotor vigilance was unaffected at altitude except for individuals with poor sleep. Significant changes were seen in physiological parameters (increased heart rate and reduced peripheral oxygen saturation). Our results may have practical implications, suggesting that individuals should practice prudence with higher ascent when performing risky activities in the first 24–36 h, even at altitudes below 3,500 m, due to an impairment of the cognitive performance that could worsen and lead to accidents.

## Introduction

There is increasing mountain attendance related to different recreational risky activities (e.g., mountaineering, skiing, and climbing), as well as for occupational purposes (e.g., mining, astrophysics) with consequently increasing accidents (Monasterio, 2005). Preserved cognitive functions, such as executive function, attention, and memory, are essential during such activities since a reduced efficiency of those abilities can provoke injury or even death in such environments. Severe acute hypoxia or anoxia was found to be related to impairment in executive function, attention, and memory (van Alem et al., 2004). Ascent to high altitude (HA) precipitates a drop in the barometric pressure and the atmospheric partial pressure of oxygen (O_2_), a condition termed as hypobaric hypoxia (HH; Taylor, 2011). The reduction of oxygen availability induces physiological changes to maintain adequate oxygen delivery, especially into the brain. The acute exposure to HH induces increased ventilation, an autoregulatory increase in cerebral blood flow and an increased oxygen extraction at the tissue/cell level. Despite these changes, a reduction in the total amount of oxygen available persists, producing a decrease in cognitive performance and different HA illnesses, especially if ascent occurs too rapidly with no acclimatization. Hornbein et al. (1989) found a slight decline in verbal and visual long-term memory and increased errors in the aphasia screening test in mountaineers exposed to altitude between 5,488 and 8,848 m.

Current results are controversial, and it is not yet clear whether cognitive abilities are selectively impaired or there is a general cognitive impairment. McMorris et al. (2017) performed a systematic review and meta-analysis on the acute effect of hypoxia on cognition. They included 18 studies, and they observed that hypoxia (both normobaric and hypobaric; arterial partial pressure of oxygen range between 35 and 89 mmHg) exerts a negative effect on cognition on both tasks investigating central executive (working memory set-shifting, updating, monitoring, inhibition, and planning) and non-executive (perception, attention, and short-term memory) functions. In a more recent review and meta-analysis, the effect of hypoxia on cognition was further confirmed, but the authors observed a selective effect: information processing seems to be enhanced (mainly in Females), whereas attention, executive function and memory impaired (Jung et al., 2020). In the 18 included studies the fraction of inspired oxygen ranged from 10 to 18%. Differences in altitude-exposure speed, duration and profile, the way of ascent, study population, cognitive tests employed, and test administration times at altitude (Li et al., 2000; Pavlicek et al., 2005; De Bels et al., 2019; Loprinzi et al., 2019) can explain discrepancies and hinder the drawing of conclusions regarding the effects of altitude on the cognition of recreationists.

Our aim was to prospectively evaluate specific cognitive functions (attention, speed processing, and decision-making) required to safely explore the mountains, as well as to work at altitude. We wanted to assess whether acute HH exposure impairs all these cognitive functions or produces selective effects on specific ones in lowlanders exposed for 3 consecutive days to an altitude of 3,269 m. At such altitude several mountain huts, winter resorts, and different occupational infrastructures are located worldwide. We also examined the correlation between cognitive performances and physiological parameters evaluated at the same timeline.

## Materials and Methods

### Participants

Participants were recruited among medical doctors or nurses participating in a mountain medicine course held in the Northern Italian Alps (Ortles-Cevedale group) at Casati hut (3,269 m). All the participants had experience in trekking. Inclusion criteria were male and female participants with an age between 18 and 60 years. Exclusion criteria was age outside that range. The study and the informed consent procedure were approved by the Institutional Review Board of Bolzano (Protocol Number 812020-BZ). The study was conducted according to the Declaration of Helsinki and reported in accordance with the START Data Reporting Guidelines for Clinical High Altitude Research (Brodmann et al., 2018).

### Study Protocol

**Figure 1:**
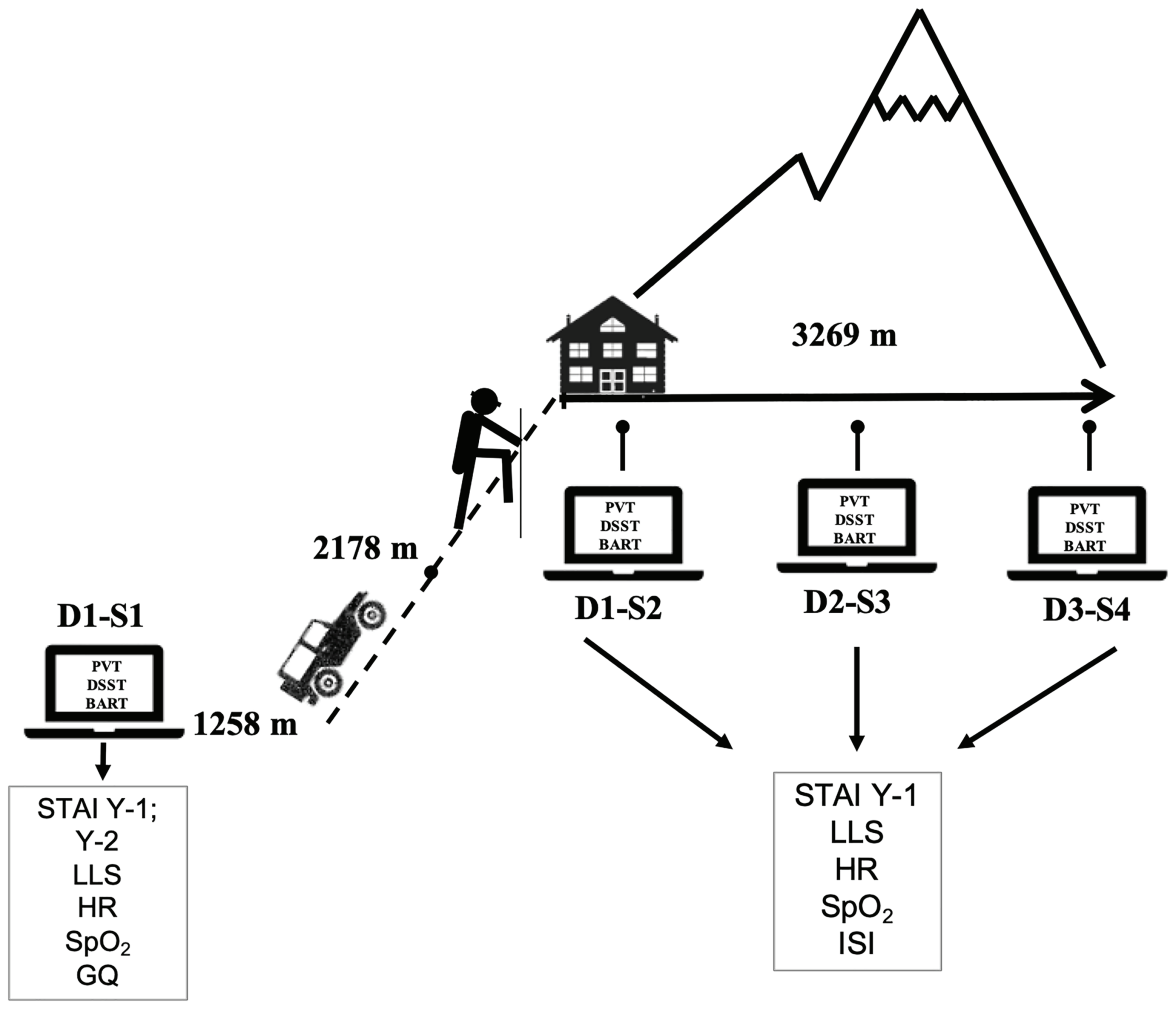
Timeline of cognitive test, questionnaires administration, and physiological-parameter recording. BART, Balloon Analogue Risk Task; D, day; DSST, Digit Symbol Substitution Test; GQ, general questionnaire; HR, heart rate; ISI, Insomnia Severity Index; LLS, Lake Louise Score; PVT, Psychomotor Vigilance Test; S, session; SpO_2_, peripheral oxygen saturation; STAI, State and Trait Anxiety Inventory (Y1-*state* and Y2-*trait*).

A longitudinal study design was performed within 3 summer days. Each participant underwent neurocognitive testing on a dedicated personal computer (PC) four times plus a familiarization session, along with the completion of several questionnaires and physiological parameters’ assessment individually and quietly (see Figure 1). All participants were asked to reach the baseline testing site staggered in groups of four individuals and at different arrival times (between 8:00 and 12:00 AM). They were initially studied in the morning for the baseline test near the trekking route (Ponte di Legno, 1,258 m; session 1, day 1, D1 S1). Participants then in groups of four drove to the parking location (2,178 m) and trekked to the Casati hut on foot along the same route (around 3:30 h). Participants were further assessed three times at altitude (3,269 m) upon arrival (session 2, day 1, D1 S2; between 6:00 and 10:00 PM), and early in the morning (between 6:00 and 8:00 AM) on the next 2 days (session 3, day 2, D2 S3, and session 4, day 3, D3 S4; see Figure 1). Before each session day (at least 2 h), participants were asked to avoid caffeine, tea, or alcohol intake. During day 2, all participants attended the mountain medicine course with minimal physical effort.

### Measures

Demographical data (age, gender, education, height, weight, altitude of residency, pregnancy, and smoking), physical activity, oral medication, or any disease (above all any neurological or psychiatric disease) were recorded. Information on staying at altitude in the 3 previous days/nights, trip >2,500 m during the last 3 months, past altitude-illness events were recorded. Physiological parameters, such as heart rate (HR) and peripheral oxygen saturation (SpO_2_), were measured in all sessions, after resting period, and in a warm and comfortable environment.

### Questionnaires on Mood, Sleep, Stress, Resilience, and Mountain Sickness

All participants completed multiple questionnaires. The administration timeline (session 1–4) of the different tests is shown in Figure 1. Anxiety and depression were evaluated using the hospital anxiety and depression scale (HADS; Zigmond and Snaith, 1983) and the State Trait Anxiety Inventory (STAI-Y1-*state* and -Y2-*trait*; Spielberger et al., 1983). *State* anxiety is a transient reaction to adverse events in a specific moment, and the *trait* anxiety is a more stable personality characteristic. Sleep quality was evaluated at baseline (session 1) using the Pittsburgh Sleep Quality Index (PSQI; Buysse et al., 1989), a questionnaire that assesses sleep quality and quantity over a month-long period. Additionally, at sessions 2, 3, and 4 the Insomnia Severity Index (ISI; Morin et al., 2011), a self-report measure that assesses participants’ perceptions of their insomnia over the previous night was used. Stress was evaluated using the 10-item version of the Perceived Stress Scale (PSS-10; Cohen and Williamson, 1988), and resilience was investigated using the Wagnild and Young’s scale (RS – 14; Wagnild and Young, 1993; Wagnild, 2009). Symptoms of acute mountain sickness (AMS) were evaluated using the Lake Louise Score (LLS; Roach et al., 2018).

### Cognitive Tests

Three different cognitive tests on a portable personal computer were employed. The brief 3-min version of the Psychomotor Vigilance Test (PVT), similar to the one reported by Basner et al. (2011), evaluated sustained attention and response time (Table 1). The Balloon Analogue Risk Task (BART; Lejuez et al., 2002) evaluated the risky decision-making. The Digit Symbol Substitution Test (DSST) measures a range of cognitive performance including speed of processing and low-level visual search, and parallel forms were used to avoid practice effects (Wechsler, 2008). Randomized test sequences were also used across the four sessions. The cognitive stimuli were presented using PsychoPy (version 3.1.0),^1^ and the software with the three cognitive tests was installed on four Eurac Research-issued laptops. To ensure that all laptops perform identically at various altitudes, laptop benchmark software (NovaBench) was run several times at different elevations.^2^ The software achieved the same scores during all tests, leading to the conclusion that a difference in altitude has no impact on the laptop’s performance.

**Table 1:** Description of the cognitive tests.

Tests	Cognitive domain	Description	Outcome measures
Digit Symbol Substitution Test *(DSST)*	Processing speed and low-level visual search	At the bottom of the screen a fix legend is presented showing blue boxes containing numbers (1–9) and on the top pairing nonsense symbols. One of the nine symbols appears randomly on the center of the screen, and the participant must select the corresponding number as quickly as possible using the keyboard numbers in a row.	**Number of correct responsesNumber of incorrect responses**
Psychomotor Vigilance Test *(PVT)*	Sustained and vigilant attention	Simple reaction time (RT) to visual stimuli that occur at random intervals presented on a screen.	**Reaction time (RT)** [ms]**Lapses:** number of omission errors or RT ≥ 355 ms**False starts:** errors of commission defined as a response without a stimulus or a RT < 100 ms
Balloon Analogue Risk Taking *(BART)*	Risky decision making	Each participant has to inflate the balloon or to cash the current virtual value of the balloon. On every pump the balloon’s size increases and can randomly explode. If a balloon explodes, the value of that balloon is lost but the previous total cashed value is unaffected.Goal is to achieve the greater reward balancing the possible loss.	**Total** amount of money **earnedTotal** count of **pumps** (only successful trials)

### Statistical Analysis

The Friedman test was used to compare LLS, STAI-Y1-*state*, HR, and SpO_2_ during all four sessions and ISI during three sessions. Pairwise comparisons were analyzed by means of the Wilcoxon signed-rank test. The parameters of the cognitive tests (BART, DSST, and PVT) were analyzed by means of generalized estimating equations (GEE), considering the following factors: session (i.e., the time of exposure to altitude), gender, age (two groups, considering the median of 26 years as cut-off), cognitive tests sequence, whether LLS was ≥3 (i.e., in the presence of headache, it is considered diagnostic for AMS) either at sessions 2 or 3, ISI (two groups, 0–7 and ≥8), SpO_2_ (two groups, <90 and ≥90%), and the interaction of session with gender. In the GEE, for BART mean earnings, BART mean pumps and PVT mean reaction time, the normal distribution and identity as link function were specified, while for DSST, the number of correct and incorrect responses and PVT number of lapses and of false starts, the specified distribution and link function were the Poisson and the logarithm, respectively; for BART, total time of test execution the gamma distribution, and logarithm as link function were specified. The Holm-Bonferroni method was used to correct the *p*-values for multiple comparisons. SPSS version 25 statistical software (IBM Corp., Armonk, NY) was used. Tests were two-sided and *p* < 0.05 was considered as statistically significant. Values are reported as mean ± standard deviation and estimates of the GEE as mean (95% confidence interval, CI).

## Results

All 36 attendants of the mountain medicine course agreed to participate and were enrolled in the study. Demographical data are shown in Table 2 (27.3 ± 4.1 year-old; 50% female; 18.9 ± 0.9 years of education). All were lowlanders and had slept at low altitude the three nights before testing; six (16.7%) slept higher than 2,500 m, and 16 (44.4%) had made a daily trip above 2,500 m in the previous 3 months. While five participants had experienced AMS in the past, no one reported high altitude cerebral oedema (HACE) or high-altitude pulmonary oedema (HAPE). Only three participants suffered neurological (one migraine) or psychiatric disturbances (one depression and one anxiety). Data about previous-month sleep and mood, stress, anxiety trait, and resilience were obtained at baseline (Table 3). The mean score at STAI-Y2-*trait* was 34.5 ± 7.7, which is in the normal range, but eight participants (22.2%) showed increased values above threshold according to age and gender (according to the Italian normative data, Pedrabissi and Santinello, 1989). Mean PSQI score was 4.4 ± 2.6, nonetheless eight participants (22.2%) were poor sleepers (mostly related to the night shifts). Mean HADS-A (anxiety; 4.2 ± 2.9) and HADS-D (depression; 1.5 ± 2.0) scores were normal (<8), but five (13.9%) participants showed a value above threshold in HADS-A while no abnormal values were observed in the HADS-D. Moderate perception of stress was present in 12 participants (33.3%), and this was referred as related to the job workload. All the participants seemed to have a good resilience (score > 64; the ability to recover quickly from difficult and potentially harmful situations; Fletcher and Sarkar, 2013). None of the participants dropped out.

**Table 2:** Demographical data (36 participants).

Features/Variables	Mean ± SD (range) or n (%)	Notes
Age, years	27.3 ± 4.1 (range 22–40)	
Females, n	18 (50%)	None pregnant
Education, years	18.9 ± 0.9 (range 16–21)	
Height, m	1.72 ± 0.09 (range 1.59–1.90)	
Weight, kg	63.8 ± 9.8 (range 48–85)	
Altitude of residence, m	238 ± 307 (range 0–1,200)	
Altitude of the 3 previous days/nights, m	228 ± 301 (range 0–1,200)	
Sleep at >2,500 m during last 3 months, n	6 (16.7%)	
Daily trip >2,500 m during last 3 months: – number of trips, n – number of participants, n	2.1 ± 4.0 (range 0–15) 16 (44.4%)	
History of altitude illnesses: – past AMS, n – past HACE, n – past HAPE, n	5 (13.9%) 0 (0.0%) 0 (0.0%)	
Physical activity: – moderate level, n – high level, n	18 (50.0%) 18 (50.0%)	
Smoker, n	5 (13.9%)	
Neurological or psychiatric disease: – migraine, n – anxiety, n – depression, n	1 (2.8%) 1 (2.8%) 1 (2.8%)	
Medication, n	11 (30.6%)	7 on demand

AMS, acute mountain sickness; HACE, high-altitude cerebral edema; HAPE, high-altitude pulmonary edema; n, number of participants/times; SD, standard deviation.

**Table 3:** Baseline questionnaires (36 participants).

Questionnaires	Mean ± SD (range) or n (%)
**STAI-Y2-*trait*** Participants with STAI-Y2 above threshold for age/gender	34.5 ± 7.7 (range 22–58) 8 (22.2%)
**PSQI** (cut-off > 5) Participants with PSQI > 5	4.4 ± 2.6 (range 1–12) 8 (22.2%)
**HADS-A** (cut-off ≥ 8) Participants with HADS-A ≥ 8	4.2 ± 2.9 (range 0–10) 5 (13.9%)
**HADS-D** (cut-off ≥ 8) Participants with HADS-D ≥ 8	1.5 ± 2.0 (range 0–7) 0 (0%)
**PSS** Participants with PSS low score (0–13) Participants with PSS moderate score (14–26)	11.2 ± 5.5 (range 3–25) 24 (66.7%) 12 (33.3%)
**RS-14**	82.5 ± 8.2 (range 65–97)

HADS, Hospital Anxiety Depression Scale; STAI-Y2-trait, State and Trait Anxiety Inventory; PSQI, Pittsburgh Sleep Quality Index; PSS, Perceived Stress Scale; RS-14, Resilience Scale 14 items.

### Physiological Parameters, Questionnaires, and LLS

**Figure 2:**
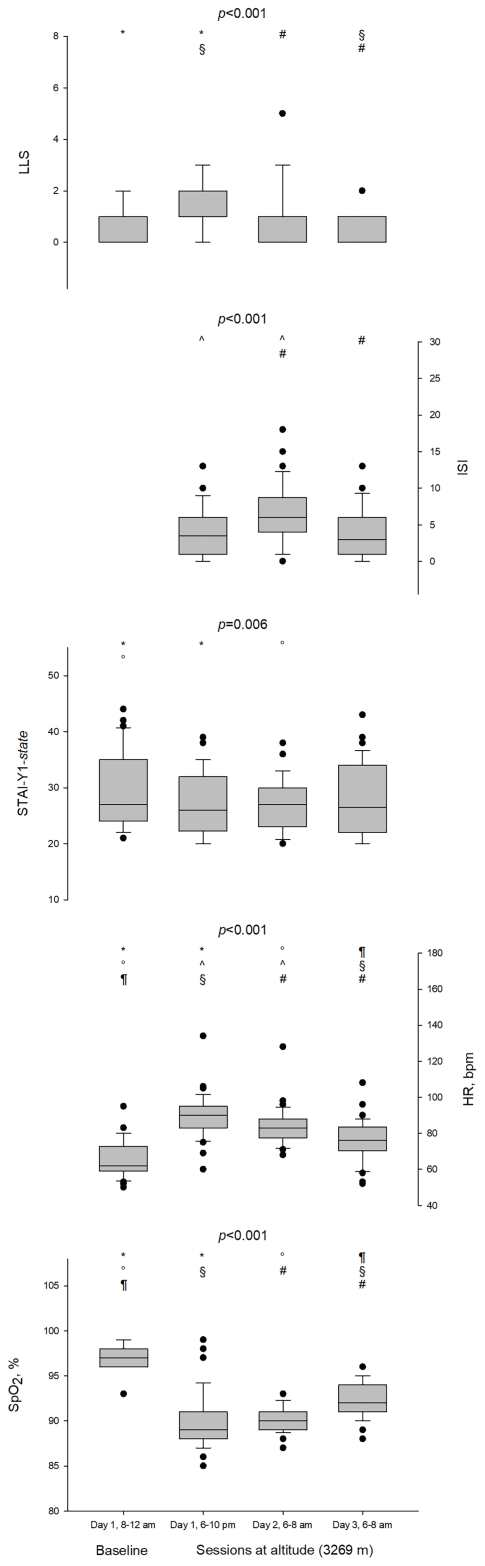
Boxplots of Lake Louise Score (LLS), Insomnia Severity Index (ISI), State and Trait Anxiety Inventory (STAI-Y1-*state*), Heart rate (HR), and peripheral oxygen saturation (SpO_2_) at baseline and sessions at altitude (3,269 m). Test performed was Friedman test. Pairwise comparisons were analyzed by means of Wilcoxon signed-rank test and the *p*-values were adjusted by means of Holm-Bonferroni correction. Statistically significant (*p* < 0.05) pairwise comparisons were denoted by the following symbols: ^*^for session 1 (day 1, 8:00–12:00 AM) vs. session 2 (day 1, 6:00–10:00 PM), ^°^for session 1 vs. session 3 (day 2, 6:00–8:00 AM), ^¶^for session 1 vs. session 4 (day 3, 6:00–8:00 AM), ^^^for session 2 vs. session 3, ^§^for session 2 vs. session 4, and ^#^for session 3 vs. session 4. bpm, beats per minute; •, outlier.

Physiological values (SpO_2_ and HR) along with the LLS, ISI, and STAI-Y1-*state* obtained across all four assessments are shown in Figure 2. SpO_2_ decreased and HR increased with acute HH exposure. LLS increased at altitude arrival (*p* = 0.015) and four participants complained of AMS (LLS 5, 3, 3, and 3) after the first night at altitude. LLS decreased after the second night at altitude returning to the baseline level (*p* < 0.001). ISI was higher after the first night at altitude (3.9 ± 3.5 vs. 6.4 ± 4.1, *p* = 0.001) but returned to the baseline level after the second night (6.4 ± 4.1 vs. 3.6 ± 3.6, *p* = 0.001). Mean values for the anxiety state measured with STAI-Y1-*state* decreased at altitude; however, the reduction was significantly different from the baseline only at sessions 2 and 3 (29.3 ± 6.6 vs. 27.0 ± 5.4, *p* = 0.033 vs. 26.9 ± 4.8; *p* = 0.032).

### Cognitive Tests (DSST, BART, and PVT)

**Figure 3:**
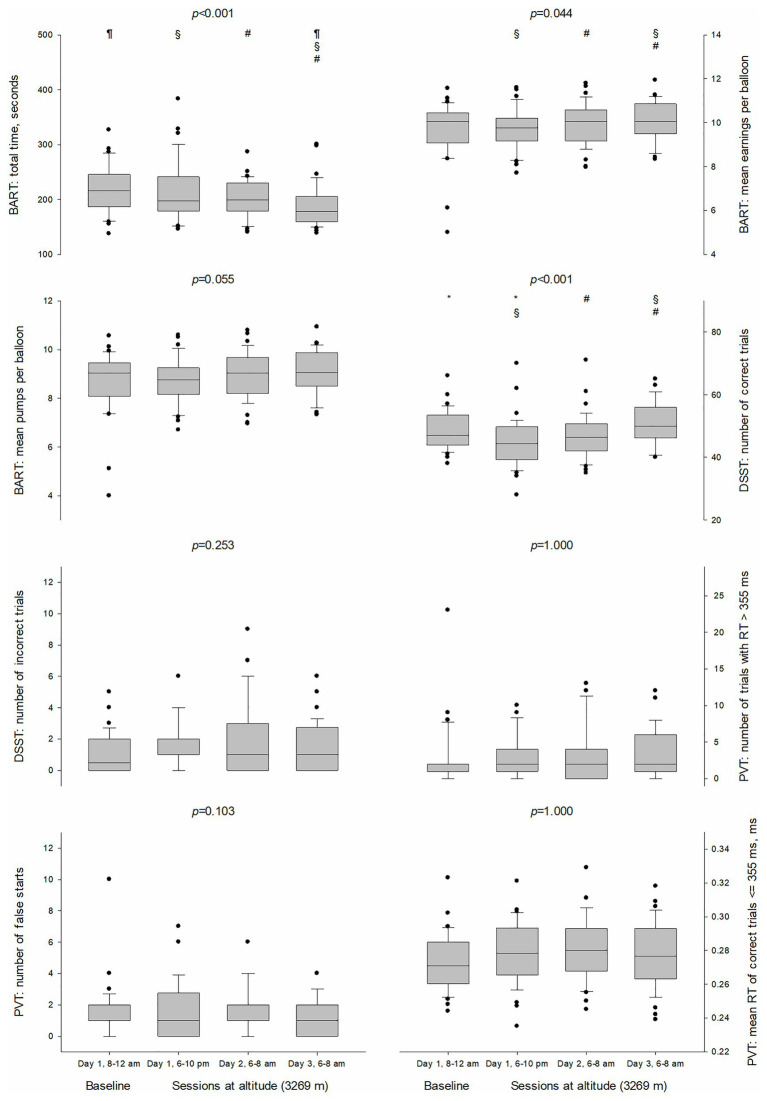
Boxplots of cognitive test parameters at baseline and sessions at altitude (3,269 m). The *p*-values were calculated by means of generalized estimating equations (GEE) and adjusted by means of Holm-Bonferroni correction. Statistically significant (*p* < 0.05) pairwise comparisons were denoted by the following symbols: ^*^for session 1 (day 1, 8:00–12:00 AM) vs. session 2 (day 1, 6:00–10:00 PM), ^¶^for session 1 vs. session 4 (day 3, 6:00–8:00 AM), ^§^for session 2 vs. session 4, and ^#^for session 3 (day 2, 6:00–8:00 AM) vs. session 4. BART, Balloon Analogue Risk Task; DSST, Digit Symbol Substitution Test; PVT, Psychomotor Vigilance Test; RT, reaction time; •, outlier.

The number of correct responses on the DSST decreased during the first 12 h at altitude (48.4 ± 6.2 vs. 44.8 ± 8.0, *p* = 0.009) and increased again after the second night at altitude (50.5 ± 6.7 in session 4, *p* < 0.001 for comparison with the session 2) (Table 4; Figure 3). GEE analysis showed no effect of altitude on the number of incorrect responses on DSST (*p* = 0.253).

**Table 4:** *P*-values of effects estimated by generalized estimating equations (GEE).

Test	Parameter	Session	Gender	Age	Test sequence	LLS ≥ 3 at session 2 or session 3	ISI	SpO_2_	Session * Gender
BART	Total time^#^	**<0.001**	1.000	1.000	1.000	1.000	1.000	0.083	1.000
	Mean earnings per balloon	**0.044**	1.000	1.000	1.000	1.000	0.668	1.000	1.000
	Mean pumps per balloon	0.055	1.000	1.000	1.000	1.000	0.790	1.000	1.000
DSST	Number of correct trials	**<0.001**	1.000	1.000	1.000	1.000	1.000	1.000	1.000
	Number of incorrect trials	0.253	1.000	1.000	0.194	1.000	0.395	0.263	0.607
PVT	Number of trials with reaction time > 355 ms	1.000	1.000	1.000	1.000	1.000	1.000	0.843	1.000
	Number of false starts	0.103	1.000	1.000	1.000	0.205	0.045	0.420	1.000
	Mean reaction time of correct trials ≤ 355 ms	1.000	0.627	0.105	1.000	1.000	1.000	1.000	1.000

An asterisk between two factors indicates the effect of interaction of the two factors. Statistically significant values are reported in bold. BART, Balloon Analogue Risk Taking; DSST, Digit Symbol Substitution Test; ISI, Insomnia Severity Index; LLS, Lake Louise Score; PVT, Psychomotor Vigilance Test; SpO_2_, peripheral oxygen saturation.

# One case excluded from the analysis because outlier.

BART total time of test execution was faster during the last session (190.4 ± 39.0 ms) in comparison to the first three (218.4 ± 44.1, 212.1 ± 52.6, and 200.9 ± 34.3 ms; *p* = 0.018, *p* = 0.001, and *p* = 0.035, respectively). BART mean earnings per balloon were slightly higher after the second night at altitude in comparison to the first session at altitude (10.1 ± 0.9 vs. 9.8 ± 0.9, *p* = 0.011) and to the session after the first night at altitude (10.1 ± 0.9 vs. 9.9 ± 0.9, *p* = 0.035). BART mean pumps per balloon did not change during the four sessions.

There was no effect of altitude on the parameters of the PVT (mean reaction time, number of lapses and number of false starts) but GEE showed an effect of ISI on the number of false starts (*p* = 0.045) as individuals with ISI higher than 7 made more false starts [1.5 (95% CI 1.0–2.2) vs. 0.9 (95% CI 0.6–1.3)].

No effect of gender on the cognitive tests was detected.

## Discussion

The main finding of this study on lowlanders after ascent to 3,269 m is that the acute exposure to HH induced impairment in oxygen saturation and produced changes in speed of processing (DSST) at arrival at altitude. There was a fairly rapid recovery since there were no more detectable effects after 36 h of exposure to HH. Psychomotor vigilance was unaffected at altitude except for individuals with poor sleep, and the BART total time of execution was faster on the last session compared to the first three, but it was not associated with clinically relevant lower performance and therefore, likely due to a learning effect.

Exposure to HH reduced SpO_2_ and increased HR due to the reduction in barometric pressure, which physiologically activates peripheral chemoreceptors and therefore sympathetic adrenergic response (Richalet, 2016). Simultaneously to physiological changes, our data provide evidence of minimal cognitive impairment after an acute exposure to altitude (3,269 m) up to 36 h in both men and women. This result is in line with other studies that showed an impaired performance on the DSST at higher altitudes and with different study designs (Evans and Witt, 1966; Berry et al., 1989; Wang et al., 2013; Hu et al., 2016). Hu et al. (2016) showed a reduced score on DSST compared to the baseline score in 100 male military participants after one night at 3650 m; after 7 days they climb to 4,400 m and a further decrease of DSST score was observed after staying for 72 h at the same altitude (4,400 m). DSST increased again after 1 and 3 months of staying at altitude (Hu et al., 2016). This finding is in agreement with our results showing a cognitive impairment already after acute HH exposure (at arrival and after around 12 h). Wang et al. (2013) evaluated the effect of acetazolamide, used to prevent AMS, on neurocognitive performance in 21 male participants flying from Xianyang (402 m) to Lhasa (3,561 m). In this randomized, double-blind, placebo-controlled study, they observed a significant decline in the acetazolamide group in the DSST performed 6 h after arrival at altitude (but not 24 or 48 h later). Similar results were obtained by Berry et al. (1989) in 20 male individuals and by Evans and Witt (1966) in 16 male individuals using a hypobaric chamber (4,500 m). Our data suggest that even at altitudes below 3,500 m, there could be an increased risk in performing demanding activities the day after arrival at altitude due to a decreased processing speed. Differently from the other studies, we enrolled both male and female, but we did not find any difference based on gender.

We also observed a quick recovery within 36 h of the initial impairment on DSST while staying at altitude, suggesting a positive effect of acclimatization. Previous studies showed an improvement of such task even with a progressive gradual ascent at altitude. Harris et al. (2009) observed a significant improvement in the DSST in 26 individuals (female and male) after 18 days of ascent to 5,100 m, or Walsh et al. (2020) in 15 individuals after 7 days of trekking to altitude (4,240 m), with impairment after exercise at higher compared to lower altitude (Walsh et al., 2020). These results may be related to the ascent profile in-agreement with the recommended guidelines to prevent altitude illnesses (Luks et al., 2019), which allows for acclimatization and prevents any neurological effects of altitude. We showed that such adaptation can occur within 2–3 days at an altitude below 3,500 m.

DSST is a fairly unspecific task that, in general, evaluates speed of processing. As with all tests, it is subject to a learning effect (improvement over repeated administrations). We used parallel forms across the repeated administration to minimize this, but the effect is not explicitly discussed in most of the studies. DSST is also sensitive to the age effect (Hoyer et al., 2004), but our sample only included relatively young and well-educated individuals. DSST is highly sensitive to detect impairment but has low specificity in determining which cognitive domain is primarily involved. In our study, the psychomotor speed and the sustained attention were also measured with the PVT; our results showed no impairment on the PVT after HH exposure. Therefore, our results suggest that the main problem of the altitude reached in our study is a reduction in general ability, namely speed of processing, so that the same tasks can be equally performed but requires a longer time of execution.

Our results showed no effects on decision-making under ambiguity. Such results are in contrast to previous studies that investigate decision-making with the BART (Heinrich et al., 2019; Pighin et al., 2020). One possible explanation is that our study sample included only health care providers (medical doctors and nurses) who engage in decision-making activities, under stress, on a daily basis. Further research should consider populations with different characteristics. Moreover, while Heinrich et al. (2019) performed an in-field study similar to us with an exposure to HH (3,800 m), Pighin et al. (2020) performed the study in a normobaric hypoxia simulated environment (3,000 m).

Our study showed preserved psychomotor vigilance after HH exposure in line with the results of other studies performed below 4,000 m (Thomas et al., 2007; De Bels et al., 2019; Heinrich et al., 2019) but is in contrast with those performed above 4,000 m (Roach et al., 2014; Davranche et al., 2016; Pun et al., 2018). However, more false starts at the PVT were observed in individuals with a worse sleep quality measured with the ISI after the first night at altitude (ISI > 7).

Four individuals complained of AMS but there was no association with worse cognitive performance compared to other individuals.

Our findings are important because a large number of lowlanders often ascend rapidly to an altitude above 3,000 m for recreational and occupational purposes. It is known that altitude illnesses can occur during travel to elevations above 2,500 m (Paralikar and Paralikar, 2010). AMS and HACE usually present detectable signs and symptoms, whereas the reduction of cognitive performance is less perceived (Neuhaus and Hinkelbein, 2014). We confirm that an impairment of selective cognitive performance can appear even after an acute exposure to 3,269 m, while other cognitive aspects are preserved (i.e., decision-making and psychomotor vigilance). Furthermore, the speed of processing impairment that was observed during the first 24 h at HA was followed by an improvement 36 h after arrival. This is an important finding that may help to improve not only the safety of mountaineers, but also of altitude workers. We suggest a resting day before planning further ascent to higher altitudes or to perform risky activities for recreational or occupational purposes to prevent not only altitude illnesses, but also the risk of accidents.

### Limitations

There are limitations worth noting. A limitation of this study was the absence of a time-matched low-altitude control group. Due to learning effects related to the repeated administration of cognitive tests, the inclusion of a control group would have been useful to isolate the altitude effect on cognitive function. Our sample was composed of relatively young individuals, and all were health-care providers, which may hamper the generalization of these findings to a broader population. However, we consider this group homogeneity selection as a strength of our study, which may broaden the application of these findings to health-care provider missions at this altitude (both rescue missions in wilderness environment reachable on foot and by helicopter). It is also uncertain whether the results would differ from those of other ethnic groups. Lastly, exhaustion was not evaluated, so we cannot say if the cognitive impairment after arrival at altitude was due solely to HH exposure or to a combination of physical effort and HH effect. Nevertheless, the persistence of the changes after a night of rest supports at least a partial effect of HH exposure per se.

## Conclusion

Our study provides evidence of a reduced processing speed in lowlanders when exposed to altitude (3,269 m) in the first 24 h at altitude. There was a fairly quick recovery since it was no longer detectable after 36 h of exposure to HH. There were no clinically relevant effects on decision-making, while psychomotor vigilance was unaffected at altitude except for individuals with poor sleep. Further investigation in populations with different ethnical background and ages are warranted to confirm this observation and potentially guide the implementation of safety procedures at altitude.

## Data Availability Statement

The raw data supporting the conclusions of this article will be made available by the authors, without undue reservation.

## Ethics Statement

The studies involving human participants were reviewed and approved by Institutional Review Board of Bolzano (Protocol Number 812020-BZ). The patients/participants provided their written informed consent to participate in this study.

## Author Contributions

MF, CP, AV, and GS contributed to the conception and design of the study. MF, AV, JK, SM-S, and GS performed the study. MF, JK, and TD organized the database. TD and MF performed the statistical analysis. MF, KH, EW, BW, MP, HB, and GS developed tools to perform the study. MF, CP, TD, JK, and GS drafted the manuscript. All authors contributed to the article and approved the submitted version.

### Conflict of Interest

The authors declare that the research was conducted in the absence of any commercial or financial relationships that could be construed as a potential conflict of interest.

## Footnotes

^1^www.psychopy.org

^2^https://novabench.com/
